# Cystic neutrophilic granulomatous mastitis: A case report and review of the literature

**DOI:** 10.3892/mi.2023.110

**Published:** 2023-09-06

**Authors:** David Gaskin, Dale Springer, Kandamaran Latha, Pamela S. Gaskin, Alain Reid

**Affiliations:** 1 Department of Pathology, Queen Elizabeth Hospital, Bridgetown, St. Michael BB11155, Barbados; 2 Faculty of Medical Sciences, University of The West Indies Cave Hill Campus, Bridgetown, St. Michael BB11000, Barbados; 3 Breast Screening Program, Barbados Cancer Society, Bridgetown, St. Michael BB11155, Barbados

**Keywords:** granulomatous mastitis, *Corynebacterium*, radiological findings, histopathology, Gram stains, differential diagnoses

## Abstract

The present study describes a case of cystic neutrophilic granulomatous mastitis. The clinical and radiological findings of the patient were consistent with idiopathic granulomatous mastitis. Cystic neutrophilic granulomatous mastitis is a rare subtype of mastitis with a distinct histological pattern that is associated with the *Corynebacterium* species. The diagnosis and treatment of cystic neutrophilic granulomatous mastitis remains a significant challenge due to the scarcity of available data. The present study describes a classic case of cystic neutrophilic granulomatous mastitis that includes clinical, radiological and histopathological findings. To the best of our knowledge, this is the first case documenting radiological findings before and after treatment. This report encourages the consideration of this entity in the differential diagnoses of mastitis.

## Introduction

Cystic neutrophilic granulomatous mastitis (CNGM) is a rare subtype of mastitis with a distinct histological pattern that is associated with the *Corynebacterium* species (1-3). The first well-documented compilation of disease associated with this species was published in 1997 by Funke *et al* (4). The association of *Corynebacterium* species with mastitis was first postulated in a 2003 review of mastitis cases by Taylor *et al* (3). *Corynebacterium* is a lipophilic Gram-positive bacillus with an affinity for adipose rich breast tissue. The organism is fastidious to growth in culture media and contemporary methods, including PCR for 16S ribosomal RNA and matrix-assisted laser desorption/ionization time-of-flight mass spectrometry (MALDI-TOF-MS) have facilitated more rapid and reliable diagnoses (5). Gene sequencing methods, such as PCR for 16S ribosomal RNA are considered the reference for the validation of MALDI-TOF-MS data.

To date, in the literature, a total of 141 cases of CNGM presenting at a mean age of 35 years have been reported since 2002 and only one of these cases was of African descent (1). To the best of our knowledge, the present study describes the first reported case of CNGM in a patient of Afro-Caribbean descent. It is suspected these cases may be underreported in the Caribbean and the scarcity of available data render accurate diagnoses and appropriate management a challenge in this population.

Cystic granulomatous mastitis predominates in the minority ethnic groups of the geographical territories with the largest published cohorts (1,6). The first case control series demonstrating an association of CNGM with the *Corynebacterium* species published in Auckland, New Zealand included predominantly Māori and Pacific islanders (3). A predilection of the disease among the Hispanic population from Central America or Mexico has been reported in North America (7). These findings are consistent with the overall epidemiological trend of granulomatous mastitis in minority ethnic groups, including Hispanic, Black, Asian and African American women (6,8). The case described herein had all the characteristic clinicopathological features of CNGM and therefore provides an ideal educational model to guide the diagnosis and management of this unusual breast pathology in a resource-limited Caribbean setting.

## Case report

### Case summary

A 32-year-old woman (gravida 2, para 2) with no known chronic medical illnesses presented to the Barbados Cancer Society-Breast Screening Programme with a 2-week history of a left breast lump and breast pain. The pain had resolved by the time of presentation, but the lump persisted. There was no associated nipple discharge or trauma to the breast. She had no previous breast surgeries. A physical examination revealed a healthy-looking young woman. There was a 6x6 cm mobile, non-tender left breast mass between the 2 to 4 o'clock positions 4 cm from the nipple. The right breast and axilla were normal.

**Figure 1:**
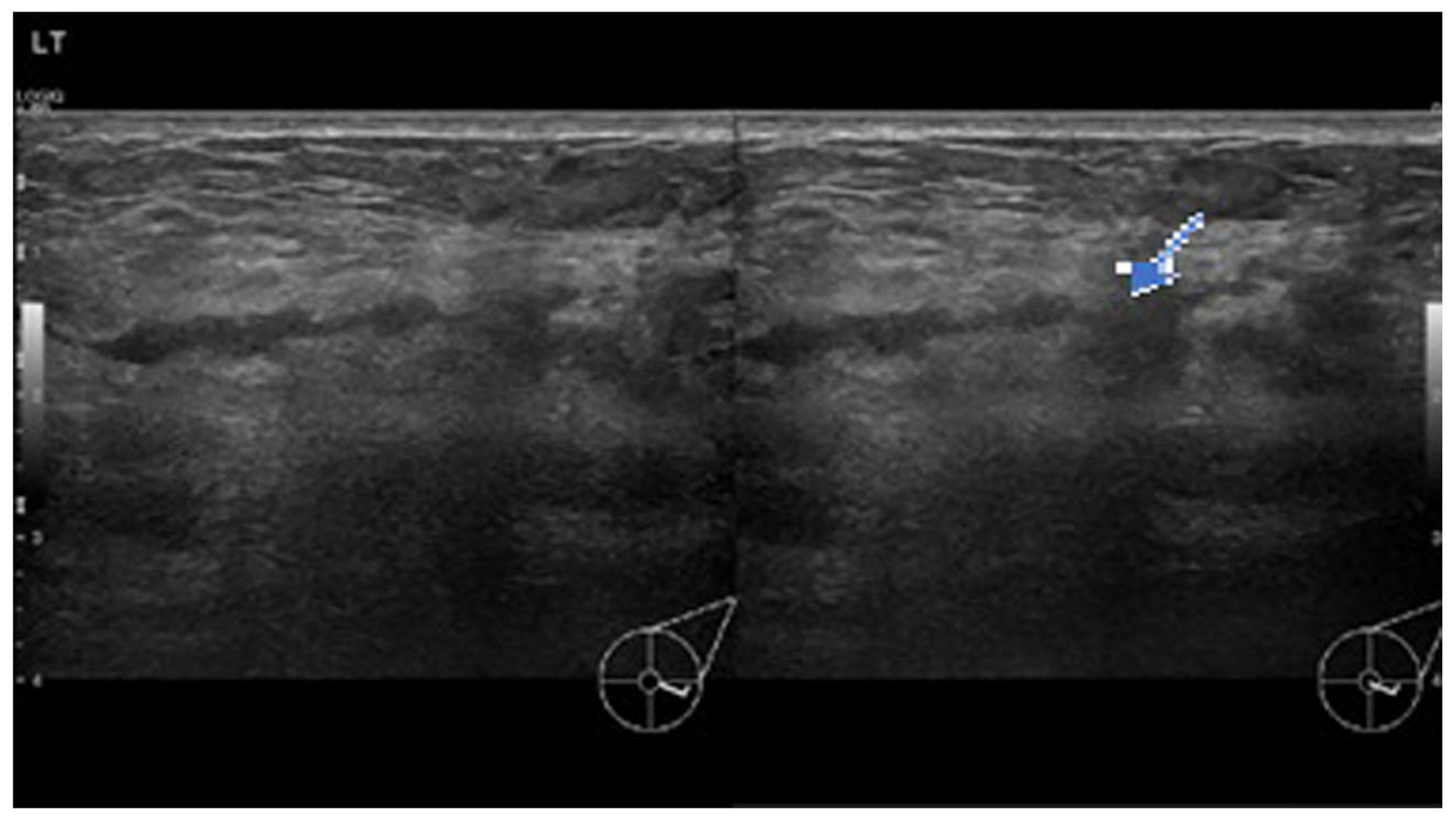
Ultrasound images illustrating transverse (right panel) and longitudinal (left panel) views of architectural distortion and dilated ducts 10 days after the initial presentation with a breast lump and pain. The arrow indicates a dilated duct surrounded by a wide area of architectural distortion.

**Figure 2:**
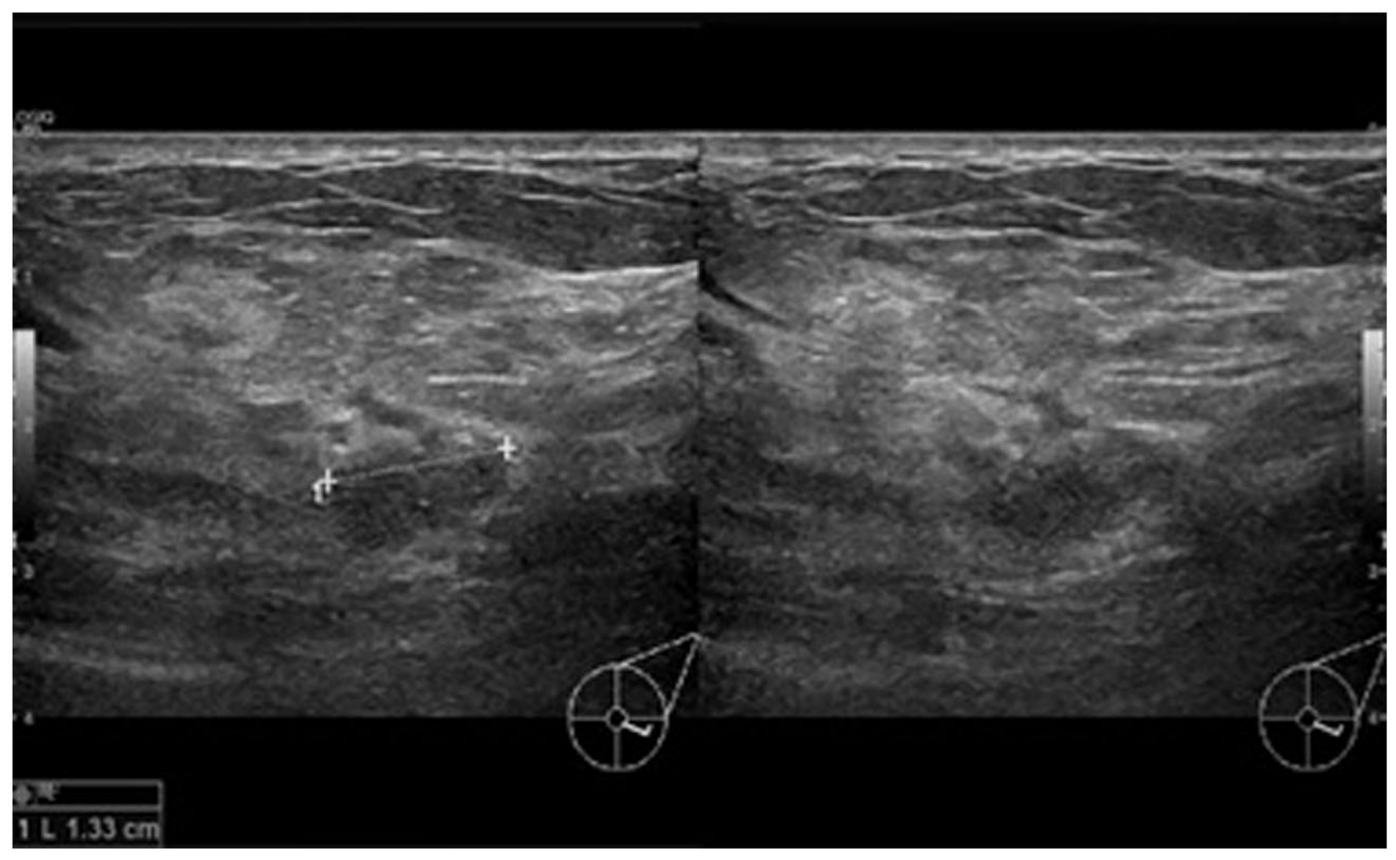
Ultrasound images illustrating transverse (right panel) and longitudinal (left panel) views of a hypoechoic mass with indistinct borders after 2 weeks of treatment with antibiotics.

**Figure 3:**
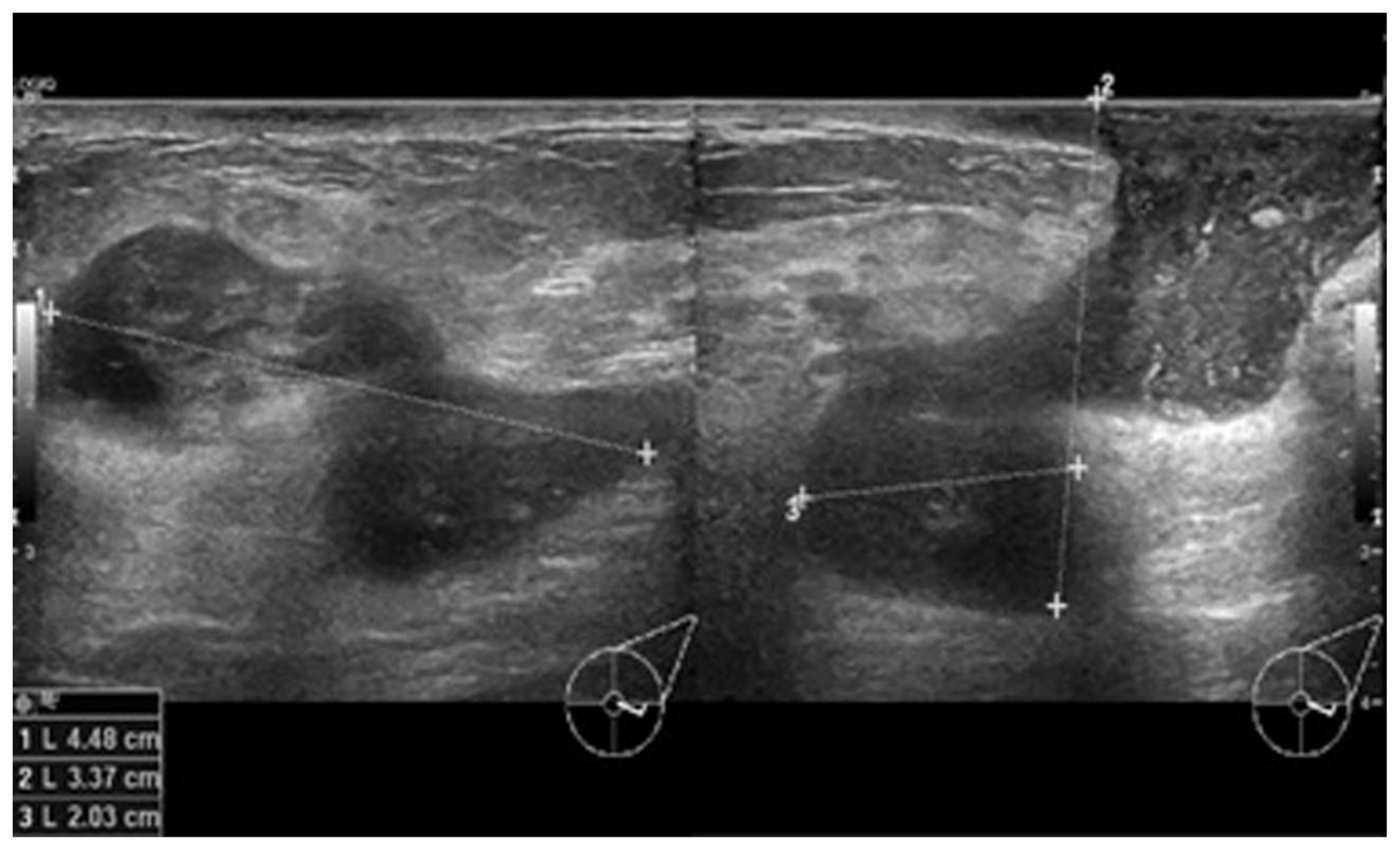
Ultrasound images illustrating transverse (right panel) and longitudinal (left panel) views 6 months after the initial diagnosis and treatment, demonstrating disease progression.

In view of the persistence of the mass, a breast ultrasound was performed. The initial examination yielded normal results. A second ultrasound repeated after 10 days (Fig. 1) revealed an area of architectural distortion with dilated ducts measuring up to 0.3 cm in diameter located at the 3 to 4 o'clock position of the left breast. There was no discrete mass or significant axillary lymphadenopathy. The findings were consistent with mastitis. The other differential diagnosis was inflammatory breast carcinoma. The patient was treated with a course of oral Augmentin (amoxicillin and clavulanic acid) and although the swelling decreased in size, it did not completely resolve. A third ultrasound (Fig. 2) after 2 weeks of treatment was suggestive of a 1.3-cm hypoechoic mass with indistinct borders at the 4 0'clock position. A decision was made to proceed with a biopsy. A repeat ultrasound (Fig. 3) was conducted after 6 months, which revealed hypoechoic tubular collections and fistulous tracts towards the skin.

### Histological findings

**Figure 4:**
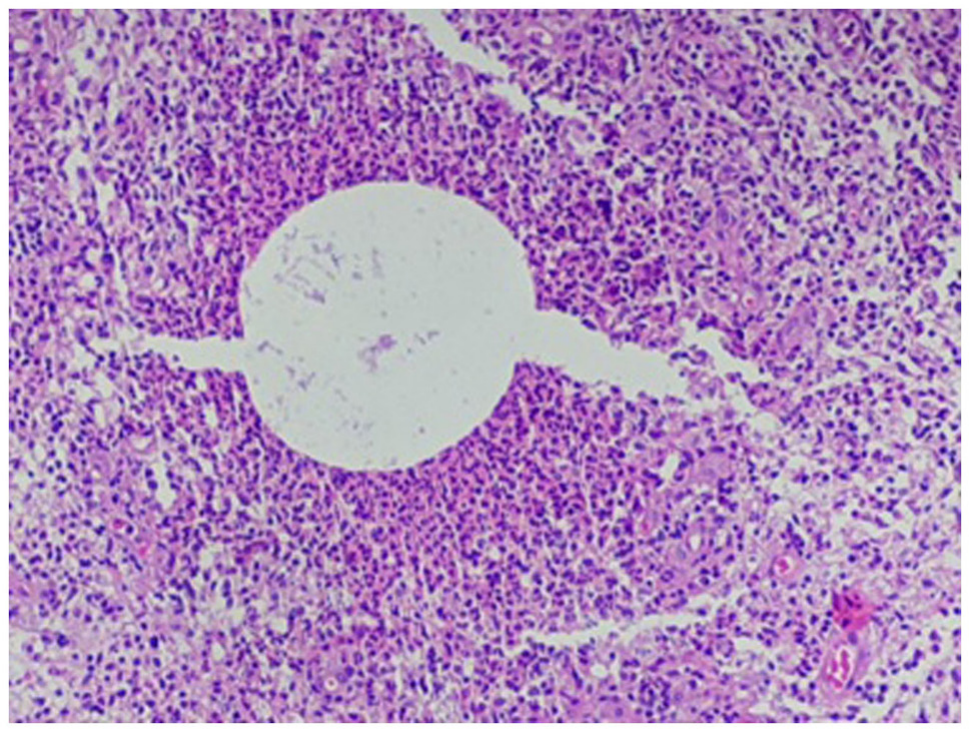
Histopathological analysis demonstrating a cystic space surrounded by neutrophils (x200 magnification).

**Figure 5:**
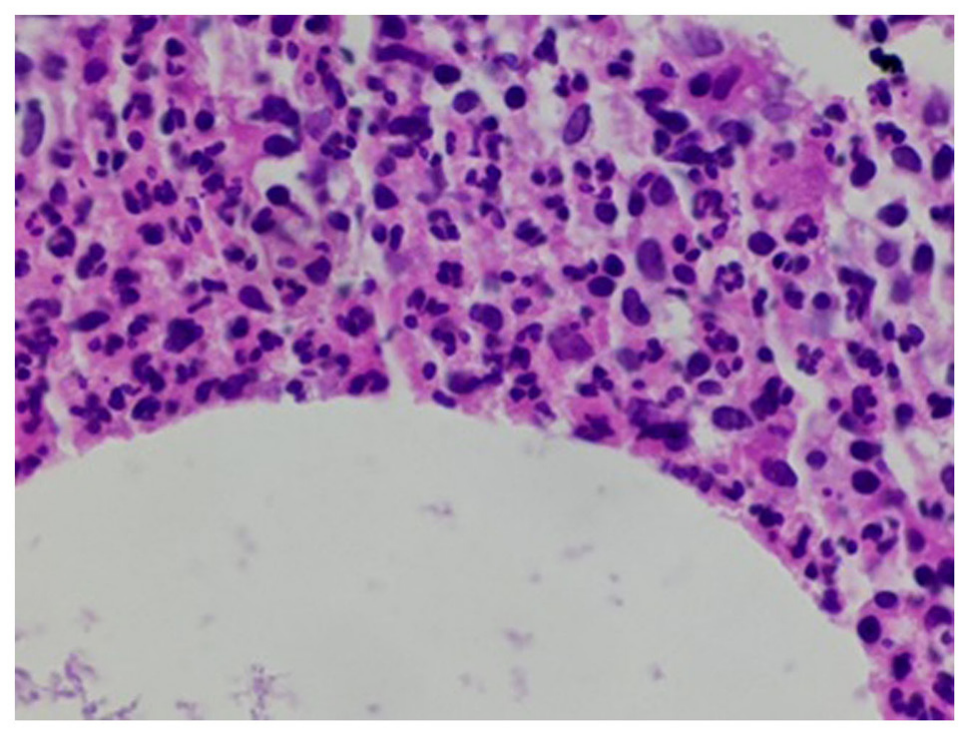
Histopathological analysis. High-power image of neutrophils surrounding the cystic space (x400 magnification).

**Figure 6:**
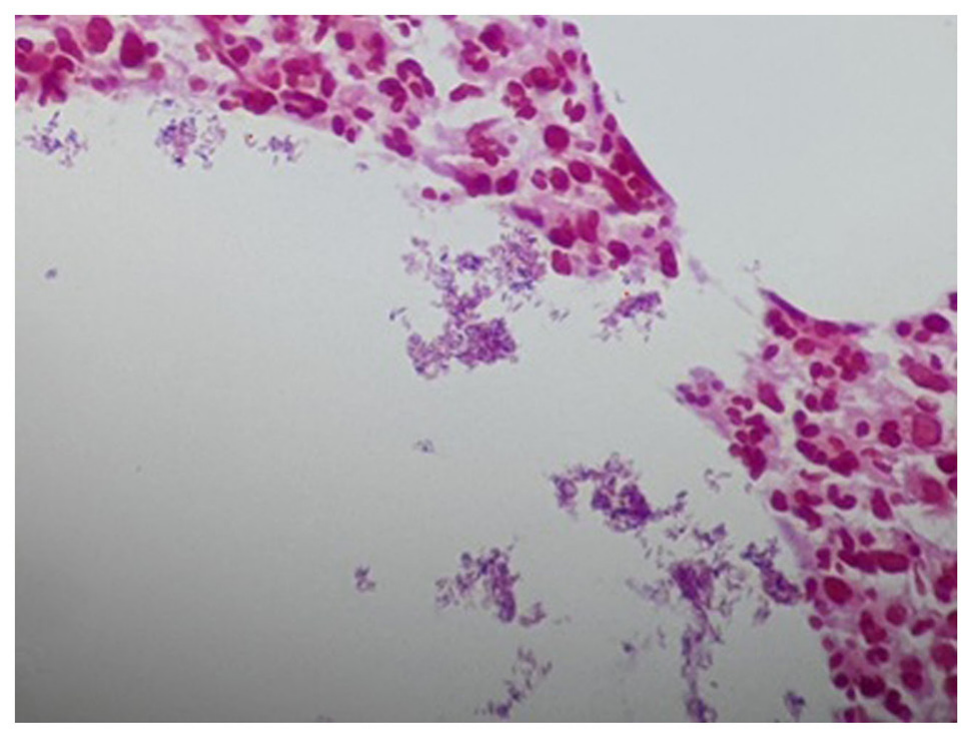
Gram stain illustrating multiple Gram-positive rods with palisading arrangement and cuneiform shapes (red dots) confined to neutrophil-rimmed cystic spaces.

Sections (4-µm-thick) were cut from paraffin-embedded tissue that was fixed in 10% neutral buffered formalin for 12 h. The Gram-Twort modified method for staining bacteria was followed and all solutions were freshly prepared by a histotechnologist according to the laboratory's standard protocol at room temperature. In summary, the tissue sections were stained with Lillie's crystal violet for 4 min and treated with Lugol's iodine solution for 4 min. The second stain was Twort's working solution for 5 min; 2% acetic acid in absolute alcohol was used for decolorization. All reagents were sourced from Stat Lab Medical Products. The stained slides were then mounted and viewed using an Olympus pathology light microscope (Olympus Corporation). The histopathological analysis of the biopsy sample using hematoxylin and eosin staining (Figs. 4 and 5) revealed cystic spaces surrounded by neutrophils. The Gram stain (Fig. 6) revealed Gram-positive blue rods within these spaces morphologically consistent with *Corynebacterium* species. The features were consistent with CNGM.

## Discussion

Granulomatous mastitis is a heterogenous group of diseases with a diverse clinical picture and association (9). It was first clearly described in 1972 by Kessler and Wolloch (10) as a lobulocentric pattern of granulomatous inflammation not associated with trauma, infection, or exogenous material. Granulomatous mastitis, as described by Kessler and Wolloch (10), is termed idiopathic granulomatous mastitis. However, there is significant overlap in the literature and a number of cases reviewed in publications on idiopathic granulomatous mastitis would meet the diagnostic criteria for CNGM (11,12).

The link between CNGM and *Corynebacterium* infection has been reported in multiple studies (1-3,7). The identification of this association places it outside the category of idiopathic granulomatous mastitis (11,12). It is fitting that this should be recognized as a distinct diagnostic entity in order to avoid overlap with other subtypes of granulomatous mastitis.

The case described herein was a nonlactating multiparous woman of 32 years of age, which fits the demographic profile for idiopathic granulomatous mastitis (12). The radiological findings in this patient included architectural distortion, with dilated ducts in the pre-treatment breast ultrasound and a hypoechoic mass with indistinct borders post-treatment with antibiotics. Radiological findings in CNGM have been sporadically reported in case reports and case series, and include a spectrum of findings (1,13). A mass is the most common radiographical feature followed by dilated ducts (1). Edema with no mass, abscesses and sinuses are also observed. The majority of cases are reported as Breast Imaging-Reporting and Data System (BIRADS) score 4 (suspicious). Malignancy is often suspected based on the finding of an irregular or ill-defined hypoechoic mass with shadowing observed on a breast ultrasound (13). The finding of a hypoechoic mass with indistinct borders in the case described in the present study is congruent with the reported radiological findings for granulomatous lobular mastitis (GLM) and CNGM (1,13,14). The literature reviewed from case reports and series has documented one radiological finding per patient. To the best of our knowledge, the present study describes the first case documenting radiological findings before and after treatment (1,13). The spectrum of findings reported for the case described herein, including dilated ducts followed by a mass and then fistulous tracts suggests a progression of the disease, despite therapy. The initial finding of dilated ducts was consistent with mastitis; however, the presence of a mass despite antibiotic therapy necessitated biopsy in this patient in order to exclude neoplasia. Inflammatory breast carcinoma was considered as a differential diagnosis for this case. Both inflammatory breast carcinoma and GLM can reveal hypoechoic masses and the ultrasound findings of the two can overlap such that biopsy of the lesion is required for a definitive diagnosis (14).

In the present study, a histopathological evaluation revealed the characteristic lobulocentric lipogranulomatous inflammation with neutrophils and multiple bacterial rods confirmed on a Gram stain. These rods exhibited a palisading arrangement with the formation of cuneiform shapes and grouping into V shapes considered to be morphologically consistent with coryneform species (13) (Fig. 6). The case described herein illustrates how well-defined light microscopic pathological features can distinguish CNGM from cases that may overlap clinically with idiopathic granulomatous mastitis.

Pathological diagnosis can be challenging, and some cases have a palisading pattern of granulomatous inflammation without the characteristic microcysts or lipid vacuoles. These cases may represent an early phase in the evolution of the disease (15). Gram stains are used to identify bacterial organisms, although these can yield false-negative results. The rate of false-negative diagnoses with a Gram stain can be reduced by focusing more closely on the lipid vacuoles that form microcysts to detect sparsely occurring Gram-positive rods. In addition, the use of multiple stains or thicker sections that increases the number of bacteria on the slide can also reduce false-negative diagnoses (15). A careful search for organisms is necessary in all cases to avoid the misclassification of CNGM as non-infectious or idiopathic and facilitate appropriate antimicrobial management for patients.

Microbial cultures would be ideal in all cases demonstrating clinical and radiological features suggestive of granulomatous mastitis. False-negative Gram stain results can occur due to the low sensitivity of histochemical stains (15). In addition, the organism is difficult to culture and additional molecular diagnostic tests, including PCR, next-generation sequencing and MALDI-TOF can improve diagnostic accuracy, if available (5,7,10,15,16).

The differential diagnoses for CNGM includes infectious and non-infectious diseases. Non-infectious causes of lipogranulomatous inflammation, including fat necrosis and silicone implants can be distinguished from CNGM by the absence of abundant neutrophils and the presence of polarizable material in giant cells (1). Autoimmune causes of necrotizing granulomatous inflammation, including granulomatosis with polyangiitis and rheumatoid arthritis have been reported in the breast. Granulomatosis with polyangiitis can be distinguished microscopically from CNGM by the presence of necrotizing vasculitis (1,17). Rheumatoid nodules presenting as granulomatous mastitis have a central area of fibrinoid necrosis palisaded by histiocytes and plasma cells. Abundant neutrophils have been described in rheumatoid nodules, but lipogranulomas are not present (18). Serology for ANCA antibodies and rheumatoid factor supports the diagnosis of these specific autoimmune causes of granulomatous mastitis (1,17,18).

Infectious causes of granulomatous mastitis, including tuberculosis can usually be excluded by the characteristic light microscopic features of the granulomatous inflammation. The granulomas of primary tuberculosis of the breast are well-formed, necrotizing and lack the neutrophils that characterize CNGM (13,19,20). Tuberculous granulomas of the breast also involve both ducts and lobules, while CNGM is confined to the lobule. Ziehl-Neelsen stain, PCR and culture would support the diagnosis of tuberculosis and other mycobacterial infections of the breast (19,20). Sarcoidosis is an idiopathic cause of granulomatous inflammation that is usually multisystemic and involves the breast in <1% of cases. Sarcoid granulomas are well-formed, typically non-necrotizing and are composed mainly of epithelioid histiocytes and Langhans giant cells (13,20,21). The role of autoimmunity or immune dysregulation in the pathogenesis and progression of CNGM is uncertain. Corticosteroids and other anti-inflammatory agents have been used in the management of CNGM; however, there is a scarcity of available evidence of their efficacy alone or in combination with antibiotic therapy (1).

Despite the wealth of evidence associating *Corynebacterium* with CNGM, a causal link has not yet been established. A proposed alternative hypothesis is that *Corynebacterium* colonizes necrotic fat parenchyma following granulomatous inflammation and is not the causative agent. However, the detection of *Corynebacterium* associated with a host immune response in deep breast parenchyma early in the course of the disease, as well as the therapeutic response to antibiotics in some cases favors an etiological link (2,3,22).

An antecedent initiating factor is not always described in cases of CNGM. However, the organism was isolated in lactating women with mastitis in one case series. In the same series, a history of trauma was documented in two non-lactating persons with CNGM from whom *Corynebacterium* was isolated (22). These findings suggest that the breach of the skin barrier secondary to trauma or breast feeding is a possible route of infection. Colonization of lactiferous ducts will allow spread to the terminal duct lobular unit, resulting in lobulocentric inflammation.

Emerging evidence suggests a potential role of other bacterial organisms in the pathogenesis of CNGM. A series of 40 cases from the Shenzhen Traditional Chinese Medicine Hospital with a CNGM-like pattern of inflammation all had negative Gram stains (16). Notably, *Corynebacterium* species was not the most common organism detected using the next-generation sequencing of paraffin-embedded tissue. Other bacterial species, including *Pseudomonas aeruginosa* were associated in these cases (16). That study, although small, provided intriguing evidence suggesting the association of organisms other than *Corynebacterium* and *Mycobacterium* with granulomatous mastitis. This finding may explain the poor response to antibiotics directed against *Corynebacterium* species in cases diagnosed only based on a Gram stain. Neither microbial culture nor molecular testing was performed in the case described herein, precluding definitive proof that *Corynebacterium* was the associated organism.

A major obstacle to the collation of data on CNGM is a lack of standardized nomenclature of the entity. Case series on idiopathic granulomatous mastitis and granulomatous mastitis include patients that would meet the diagnostic criteria for CNGM (11,23,24). A standardization of the diagnostic nomenclature will enable more comprehensive research to develop protocols for the diagnosis and treatment of this entity. The application of the term CNGM should thus perhaps only be used in cases that exhibit the histomorphological pattern and evidence of infection with Gram-positive rods morphologically consistent with *Corynebacterium* species (24). An effort should be made to culture the organism or conduct ancillary molecular tests for confirmation, if possible.

The present study describes a rare classic case of CNGM occurring in a woman of Afro-Caribbean descent. The clinicopathological features were characteristic, and Gram-positive bacilli were identified on a Gram stain. This case contributes to the increasing evidence that CNGM is a histomorphologically distinct entity associated with bacterial infection. The pathologist can play a critical role in patient management by recognizing the pattern of inflammation and requesting the appropriate histochemical stains. The finding of an infectious organism can direct antimicrobial therapy.

*Corynebacterium* species have been confirmed in numerous reported cases (13,22,24) with the pattern of neutrophilic granulomatous inflammation and the cuneiform configuration of Gram-positive bacilli demonstrated in the case described herein. In light of these histomorphological findings, *Corynebacterium* may be considered to be the most probable infectious etiology. Ideally, the authors of the present study would have liked to obtain culture or molecular testing as supportive evidence that the Gram-positive rods identified in this case were indeed *Corynebacterium*. However, these more specific tests were costly and were not locally available.

To the best of our knowledge, the present study reports the first case of CNGM in the English-speaking Caribbean. The identification of bacterial organisms in the present case underscores the widely reported role of bacterial infection in the etiopathogenesis of this entity. The treatment of this disease remains a significant challenge due to the scarcity of available data for effective treatment protocols. This report encourages the consideration of this entity in the differential diagnoses of mastitis among Afro-Caribbeans.

## Data Availability

The datasets used and/or analyzed during the current study are available from the corresponding author on reasonable request.
